# Lightweight Design Method for Micromanufacturing Systems Based on Multi-Objective Optimization

**DOI:** 10.3390/mi16091032

**Published:** 2025-09-09

**Authors:** Shan Li, Seyed Hamed Hashemi Sohi

**Affiliations:** 1 School of Mechanical Manufacturing and Energy Engineering, Mapúa University, Manila 1002, Philippines; shhashemisohi@mapua.edu.ph; 2 School of Mechanical and Electrical Engineering, Zhengzhou Business University, Zhengzhou 451200, China; 3 School of Graduate Studies, Mapúa University, Manila 1002, Philippines; 4 Zhengzhou Intelligent Electromechanical Engineering Research Center, Zhengzhou Business University, Zhengzhou 451200, China

**Keywords:** topology optimization, lightweight design, response surface methodology, micromanufacturing systems, dynamic stiffness, structural optimization, natural frequency, sensitivity analysis

## Abstract

This study proposes a multi-stage collaborative design framework integrating sensitivity analysis, response surface methodology (RSM), and topology optimization for synergistic lightweighting and performance enhancement of micromanufacturing systems using ultra-precision computer numerical control (CNC) machine tools. Overall sensitivity analysis identified the base and column as stiffness-critical components, while the spindle box exhibited significant weight-reduction potential. Using spindle box wall and bottom thickness as variables, RSM models for mass and stress were constructed. Multi-objective optimization via a genetic clustering algorithm achieved a 57.2% (590 kg) weight reduction under stress constraints (<45 MPa). Subsequent variable-density topology optimization (SIMP model) reconfigured the rib layouts of the base and column under volume constraints, reducing their weights by 38.5% (2844 kg) and 41.5% (1292 kg), respectively. Whole-machine validation showed that maximum static deformation decreased from 0.17 mm to 0.09 mm, maximum stress reduced from 58 MPa to 35 MPa, and first-order natural frequency increased from 50.68 Hz to 84.08 Hz, significantly enhancing dynamic stiffness. Cumulative weight reduction exceeded 3000 kg, achieving a balance between lightweighting and static/dynamic performance improvement. This work provides an effective engineering pathway for a structural design of high-end micromanufacturing systems.

## 1. Introduction

With the continuous growth in the demand for high-performance processing equipment in microfabrication fields such as precision optical components and micro-mold processing, the structural performance of ultra-precision CNC machines has become a key factor affecting their processing accuracy and operational stability. During operation, micromanufacturing systems bear complex multi-axis loads and need to meet multiple performance indicators such as high rigidity, rapid response, and high positioning accuracy. Traditional methods to enhance rigidity often involve increasing the structural size or wall thickness, which can enhance rigidity, but also bring problems such as increased weight, increased energy consumption, and delayed response [1]. Especially in five-axis structures, an uneven weight distribution can easily cause vibration and positioning errors [2]. The pursuit of high performance and low energy consumption has made lightweight design a critical research focus in advanced manufacturing [3,4].

With the rapid development of intelligent manufacturing, achieving lightweight structures while ensuring sufficient rigidity and dynamic performance has become crucial in micromanufacturing system optimization. Parameter optimization methods, particularly those combined with sensitivity analysis, play a fundamental role in identifying the key structural parameters necessary for performance improvement. Wang et al. optimized geometric parameters using sensitivity analysis to reduce structural mass [5], while Yang et al. employed the EFAST method to identify error sources in gear processing [6]. Other contributions include Ji et al.’s combination of the Morris method with intelligent algorithms for stiffness enhancement [7], Ellinger et al.’s demonstration of sensitivity analysis’ superiority over traditional methods [8], and Hao et al.’s integration of a response surface method with sensitivity analysis for balancing stiffness and energy efficiency [9].

The response surface method has proven to be highly effective for lightweight optimization due to its efficiency in establishing performance-variable relationships. Wei et al. developed RSM models for CNC gantry machine beds, achieving significant reductions in deformation and improvements in modal frequency [10]. Wu et al. proposed a multi-objective optimization process using a Taguchi design and finite element analysis [11], while Huang focused on turntable optimization using RSM [12]. Hernandez-Vazquez et al. further demonstrated the effectiveness of combining RSM with sensitivity analysis for stiffness and frequency optimization [13].

Topology optimization has emerged as a powerful approach for weight reduction in micromanufacturing systems. Lin and Chang achieved substantial stiffness improvements using topology reconstruction [3], and Behera et al. combined parameter optimization with topology design to reduce weight while improving dynamic response [14]. Liu et al. employed sensitivity analysis and multi-objective methods for cross-brace optimization [15], while Sun et al. emphasized enhanced performance through the integration of parameter and topology optimization [16]. Recent research has progressed toward multi-method integration: Fu et al. further confirmed the effectiveness of topology optimization in enhancing the dynamic performance of microresonators [17], Jiang et al. combined Kriging models with MDO [18], Li et al. developing reduced-order model-based sensitivity analysis [19], and other researchers addressed complex optimization scenarios using advanced methodologies [20,21,22,23].

Based on the existing finite element model and previous research foundations, this study proposes a multi-stage collaborative design framework that integrates sensitivity analysis, response surface methodology, and topology optimization. This research aims to (1) identify the key components with weight-reduction potential using sensitivity analysis; (2) utilize RSM for multi-objective optimization of the spindle box; (3) employ topology optimization to restructure the base and column; and (4) verify the overall performance improvement using whole-machine simulation analysis. This work provides an effective engineering pathway for the structural design of high-end micromanufacturing systems, with potential applications in micro-feature machining, which requires high dynamic stiffness.

## 2. Methodology

### 2.1. Foundation of Research

**Figure 1:**
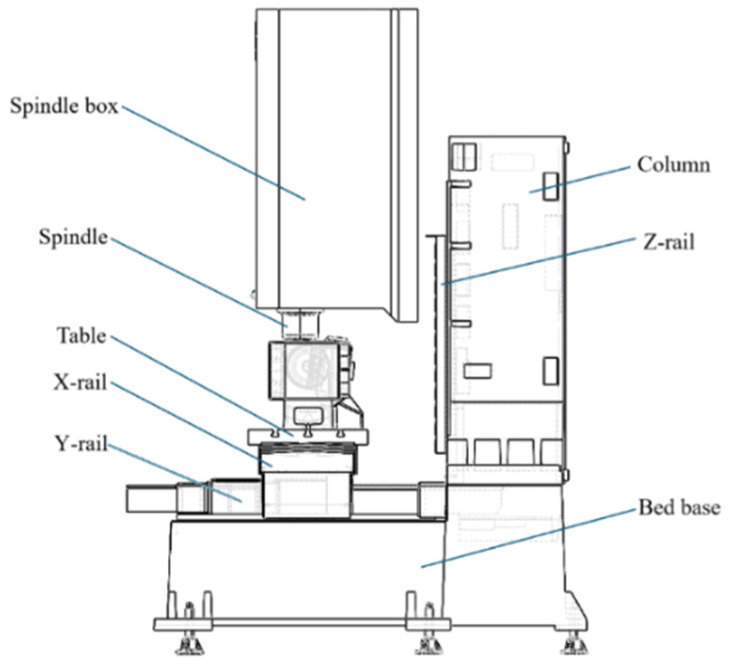
Structure of the ultra-precise five-axis CNC micromanufacturing system.

**Figure 2:**
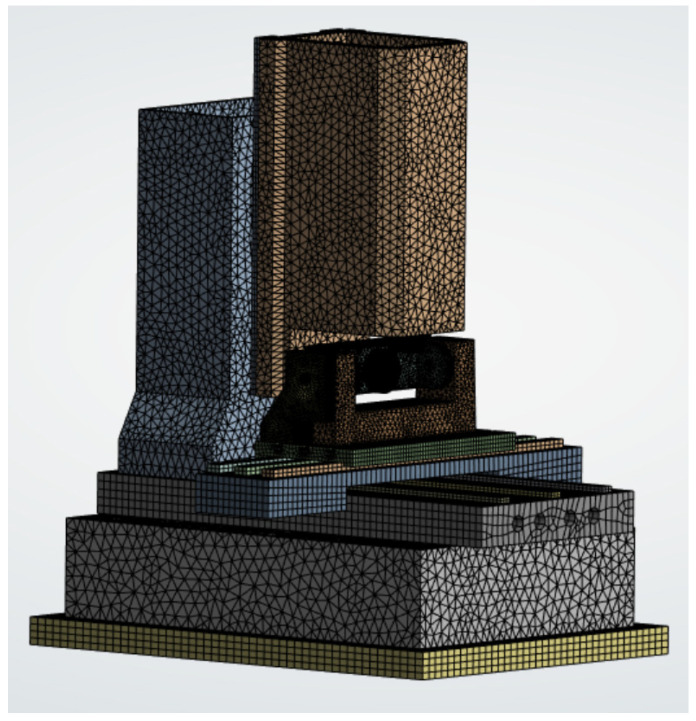
Finite element model of the machine.

Before the commencement of this study, the authors completed the finite element modeling and static/dynamic characteristic analysis of the ultra-precise five-axis CNC micromanufacturing system (Figure 1), the finite element model of the machine can be seen from Figure 2. The model was established based on the actual structural dimensions and working conditions. Using static analysis, the stress concentration areas were identified, and the key role of the spindle box in the deformation and stiffness distribution of the entire machine was clarified. Modal analysis indicated that the base and the column had a significant impact on the overall vibration performance. On this basis, the initial parameter optimization of the spindle box was completed, providing the necessary modeling conditions and analysis basis for further multi-objective weight reduction optimization in this study. It will now be used as the foundation for subsequent optimization design work in this study.

In the previous study, the boundary conditions were explicitly defined as follows: (1) Constraint conditions: The machine tool’s foot was subject to complete fixed constraints, simulating the bolt connection with the ground. (2) Load conditions: Overall application of gravitational acceleration, and, based on the empirical data from aerospace alloy processing, the maximum thrusts of the *X*-axis, *Y*-axis, and *Z*-axis were 11,343 N, 11,343 N, and 18,683 N, respectively, and these three forces were applied to the end of the spindle according to the corresponding X, Y, and Z directions so as to simulate the most hazardous conditions of the actual machining process. Based on the static and dynamic simulation results of the entire micromanufacturing system, the weak components—the thickness of the base (P_1_), the thickness of the column (P_2_), and the thickness of the spindle box (P_3_)—were optimized (the results were P_1_ = 60 mm, P_2_ = 120 mm, and P_3_ = 60 mm). The stiffness of the entire machine was significantly improved, with the maximum deformation decreasing from 0.17 mm to 0.039 mm and the first-order natural frequency increasing from 50.7 Hz to 85.5 Hz. However, this solution led to an increase of 24.8% in the weight of the spindle box, causing problems such as increased inertial load and increased energy consumption. Therefore, in the following sections of this paper, a further collaborative optimization design scheme is proposed.

### 2.2. Structural Optimization of the Spindle Box

#### 2.2.1. Sensitivity Analysis Identifies the Key Design Parameters

In order to identify the design parameters in the entire machine structure that have a significant impact on static deformation, this paper takes the thickness of the base (P_1_), the thickness of the column (P_2_), and the thickness of the spindle box (P_3_) as the initial design variables. The thickness of the base (P_1_), the column (P_2_), and the spindle box (P_3_) were selected as the initial design variables for the overall sensitivity analysis because they represent the dominant macro-geometric parameters controlling overall structural mass and stiffness, based on engineering precedent and the findings from our preliminary FEA. On the ANSYS DesignXplorer platform (ANSYS Workbench 2024 R2), the Spearman rank correlation coefficient method is used for overall sensitivity analysis to find out the influence of component design parameters on the static and dynamic mechanical performance of the structure. The range of change for the three variables was set according to the structural dimensions, and the maximum total deformation was used as the response index. The linear correlation coefficient method was applied to evaluate the strength of the relationship between the variables and the response.

**Figure 3:**
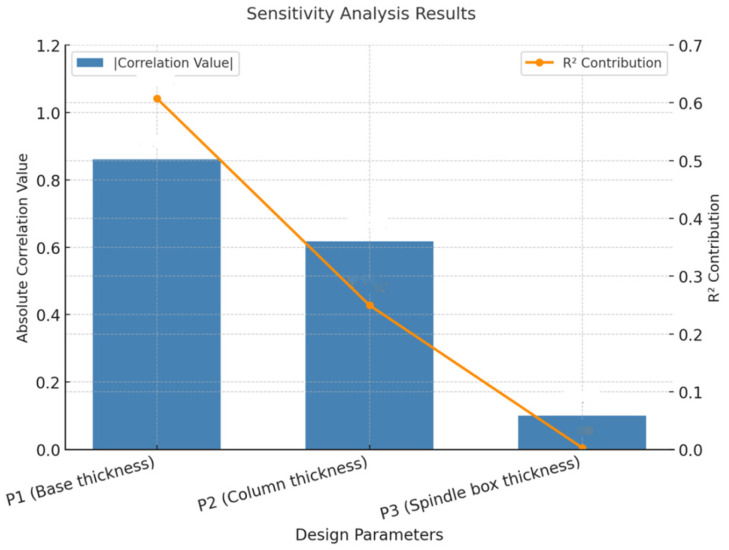
The results of the sensitivity analysis.

The sensitivity analysis results are shown in Figure 3, which plots both the absolute correlation coefficients and the R^2^ contributions. P_1_ has a strong negative correlation with the maximum deformation of the entire machine, with a correlation coefficient value of approximately −0.861, and also demonstrates the highest R^2^ contribution (0.608), confirming it as the most influential parameter. P_2_ shows a moderate negative correlation (correlation coefficient: −0.617,) with a significant R^2^ contribution (0.250). The correlation of P_3_ is relatively low, with a correlation coefficient value of approximately −0.101 and a negligible R^2^ contribution (0.003). The results indicate that the base and the column have a higher sensitivity to the deformation of the entire machine, suggesting that they play a dominant role in the structural stiffness; while the sensitivity of the spindle box is lower, and the change in its thickness has a negligible effect on the mechanical performance. Therefore, the spindle box has significant space for weight reduction without affecting the rigidity of the micromanufacturing system, and lightweight design will be carried out for it in the future.

#### 2.2.2. Response Surface Modeling and Multi-Objective Optimization of the Spindle Box

To enhance the efficiency of the weight reduction design for the local structure of the spindle box, based on the previous parameter refinement, this paper selects the wall thickness of the spindle box (P_4_) and the bottom thickness (P_5_) as the optimization design variables. The main performance indicators are structural quality and maximum equivalent stress. A surrogate model is constructed for multi-objective optimization analysis. The basic flow of response surface modeling includes sample point design, finite element simulation response extraction, mathematical model fitting, optimization solution, etc.

**Figure 4:**
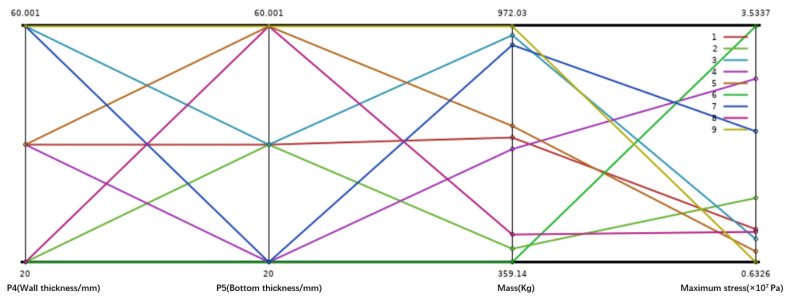
Sample point distribution map of design variables for the spindle box.

The sample points were generated using a Central Composite Design (CCD). For the setting of variable ranges, considering the actual conditions of the machine tool, the ranges of P_4_ and P_5_ are both set between 20 and 60 mm. Then, a certain number of design points are formed during the experimental design stage, thereby generating a sample space, as shown in Figure 4. Each line in the figure represents a design sample. Each sample point is subjected to finite element analysis in ANSYS to obtain the corresponding quality and maximum equivalent stress results. The corresponding ranges of the output responses were determined through preliminary simulations: the mass varied between approximately 359 kg and 972 kg, while the maximum equivalent stress ranged from about 6 MPa to 35 MPa.

**Figure 5:**
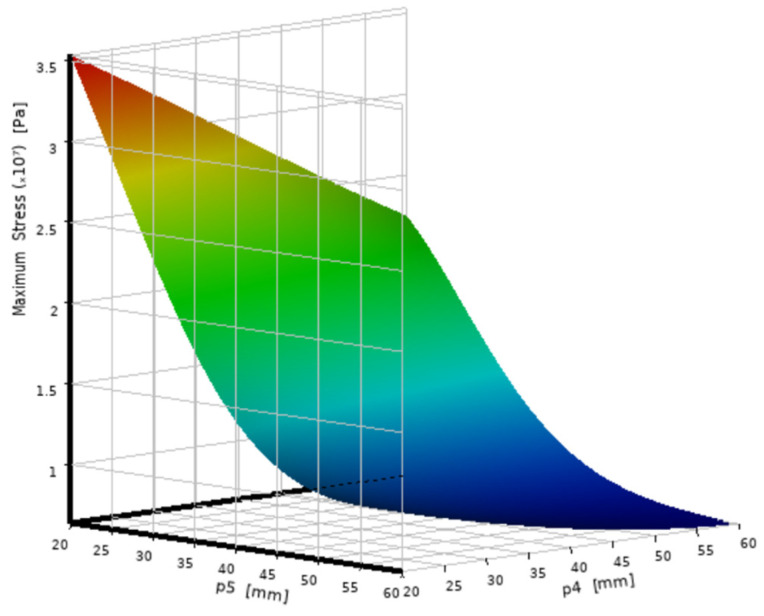
Stress response characteristic diagram.

**Figure 6:**
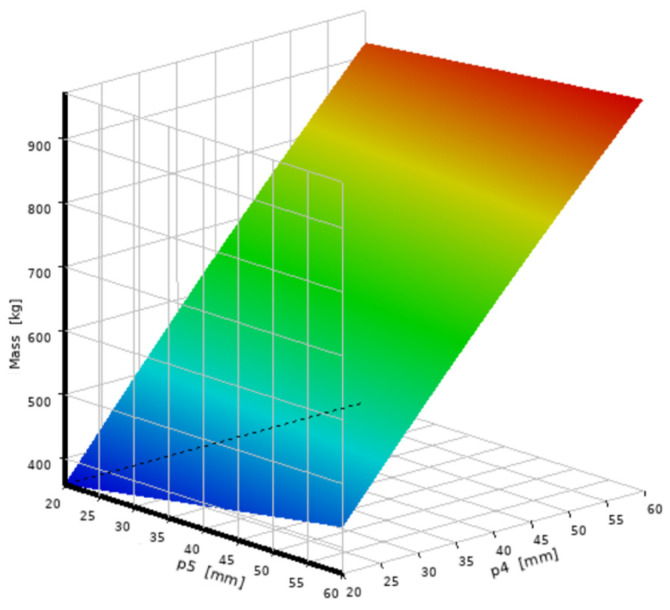
Quality response characteristic diagram.

Based on the second-order response surface model constructed from the DOE samples, the relationship between the design variables and each response is visualized in Figure 5 and Figure 6. These 3D surface plots were generated using ANSYS DesignXplorer to represent the fitted surrogate model. From the simulation response trend, Figure 5 shows that the stress gradually decreases as P_4_ and P_5_ increase. Among them, the influence of wall thickness P_4_ on stress is particularly significant. However, after P_4_ exceeds 25 mm, the trend of stress decline slows down, and the marginal optimization benefit weakens. Figure 6 indicates that quality has a linear positive correlation with both P_4_ and P_5_, and the bottom thickness P_5_ has a more significant impact on the total quality.

Based on this, the optimization objective was set as minimizing the quality, and the constraint condition was set as the maximum stress being less than 45 MPa. The genetic aggregation algorithm was used for multi-objective optimization. The Pareto solution set was adopted to reflect the trade-off relationship between quality and stress under different combinations of design variables. Finally, P_4_ = 20 mm and P_5_ = 30 mm were selected as the optimal solution combination. The simulation results show that, under the constraint condition of the maximum stress being less than 45 MPa, the maximum stress was reduced to 18.09 MPa and the structural quality of the spindle box was reduced by approximately 57.2% compared to the original design, significantly improving the lightweighting level and ensuring the structural safety margin.

Although the sample size used in this section is relatively small, the response surface model established has excellent fitting performance within the parameter range, which can provide effective support for multi-objective optimization. The research results further verify the practicality of the RSM method in the weight reduction design of complex structures.

### 2.3. Structural Reconstruction of the Base and Column Based on Topological Optimization

To achieve the coordinated optimization of stiffness and mass for high-sensitivity components (base and column), the variable density topology optimization method was employed for material redistribution. This method is based on the isotropic material penalty model (SIMP), and, through iterative solution, it maximizes the structural stiffness while minimizing the mass. Its mathematical model is expressed as follows:

(1) $$\rho_{e}\left(e=1,2,\ldots ,N\right),$$

The objective function is expressed as follows:

(2) $$\min c(\rho )=U^{T}KU=\sum_{e=1}^{N}\rho_{e}^{p}u_{e}^{T}k_{0}u_{e},$$

(3) $$s.t.\frac{V(\rho )}{V_{0}}\leq f_{v},$$

(4) KU=F,

(5) $$0<\rho_{\min}\leq \rho_{e}\leq 1,$$

In the above equation, $\rho_{e}$ represents the density design variable of the *e*-th element, *N* represents the total number of elements in the finite element mesh, *p* represents the penalty factor, c(ρ) represents the flexibility of the structure—that is, the total deformation—*V* represents the material volume, and $f_{v}$ represents the volume fraction constraint.

The optimization problem was solved using the Method of Moving Asymptotes (MMA). The convergence was assumed when the change in the objective function (compliance) was less than 0.1% over 10 consecutive iterations. A penalty factor of *p* = 3 was used for the SIMP model to promote a clear, solid-void material distribution.

#### 2.3.1. Topological Optimization Design of the Base

**Figure 7:**
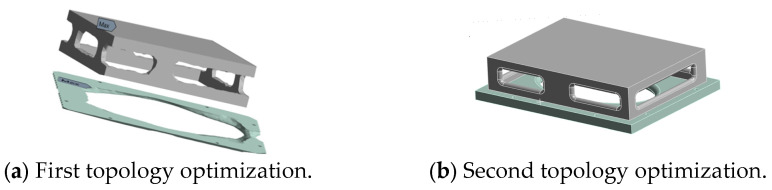
The topological optimization result of the base plate.

Firstly, the topology optimization method based on the variable density approach (SIMP model) was applied to reconfigure the base structure. The design domain covered the main body of the base, while the bolt installation surface was set as the non-design domain to ensure the assembly function. The optimization objective was defined as a dual-constraint problem: under the condition of meeting the volume fraction constraint ($f_{v}\leq 0.7$), the topology optimization was carried out with the goal of minimizing the flexibility and maximizing the natural frequency. The horizontal set algorithm was used for iterative solution, with the penalty factor set as *ρ* = 3. The first optimization result of the base is shown in Figure 7a. Then, based on the optimized shape, the reconfiguration design was carried out. The reconfigured base is shown in Figure 7b.

#### 2.3.2. Topological Optimization Design of the Column

**Figure 8:**
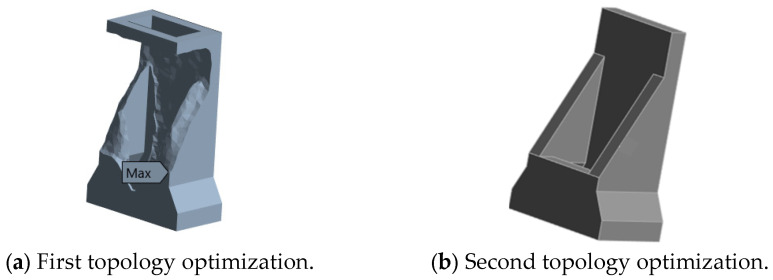
The topological optimization result of the column.

The optimization of the column was carried out in the same way, with the focus on addressing the synergy issue between the Y-direction bending stiffness and lightweighting. The design domain included the main body of the column, while the guide rail installation surface was retained as the non-design domain. XZ plane symmetry constraints were applied to suppress the unbalanced load vibration. The objective function was set to maximize the Y-direction bending stiffness, while satisfying the constraint of a 40% volume reduction. The first optimization result of the column is shown in Figure 8a, and the reconstructed designed column is shown in Figure 8b. The strengthened ribs generated by the optimization exhibit a gradient feature: the rib thickness gradually changes from 40 mm at the bottom to 25 mm at the top. This layout avoids the stress concentration phenomenon of traditional vertical rib plates through uniform stress distribution, and also suppresses the local modal vibration caused by the accumulation of mass.

## 3. Performance Comparison Analysis Before and After Optimization

In order to achieve the structural lightweighting of the micromanufacturing system while maintaining its rigidity and dynamic performance, we conducted parameter optimization and topology optimization design for the key weak components identified in the overall structure: the spindle box, the base, and the column. The performance comparison analysis of stress, deformation, and weight before and after optimization is presented below.

### 3.1. Optimization Effect of Weak Parts

Firstly, in terms of stress, the three major components all achieved significant improvements using different optimization methods. As shown in Table 1, the maximum equivalent stress of the spindle box before optimization was 61.79 MPa. Through the design parameter refinement and multi-objective optimization using the response surface method, the stress decreased to 18.09 MPa after optimization, a reduction of 70.7%, significantly enhancing the structural safety margin. Under the topological optimization, the maximum stress of the base was reduced from 4.68 MPa to 4.31 MPa, effectively alleviating the stress concentration phenomenon in the key load-bearing area. After the column optimization, the stress decreased from 10.16 MPa to 4.17 MPa, indicating that the optimization scheme maintained the structural rigidity while achieving good control of the bearing capacity.

Secondly, from the perspective of deformation control, after the optimization of the three components, all achieved effective improvements while maintaining enhanced stiffness. As shown in Table 2, the maximum deformation of the spindle box decreased from 0.166 mm to 0.091 mm, the maximum deformation of the base reduced from 0.019 mm to 0.008 mm, and the deformation value of the column decreased from 0.123 mm to 0.067 mm. Although the absolute numerical changes are not significant, under the requirements of precision processing, such micrometer-level stiffness improvements are crucial for the positioning accuracy and processing stability of the entire machine.

Finally, in terms of weight, all components have achieved significant weight reduction. As shown in Table 3, the spindle box was optimized using certain parameters, and its weight decreased from 1379 kg to 590 kg, a reduction of 57.2%. The base was optimized using topology, and its weight was reduced from 4624 kg to 2844 kg, a reduction of 38.5%, while the weight of the column was reduced from 2210 kg to 1292 kg, a reduction of 41.5%. The overall weight decreased significantly by more than 3000 kg, providing a strong support for the improvement of the overall dynamic response capability, the reduction in driving energy consumption, and the increase in processing efficiency.

### 3.2. The Optimization Effect of the Whole Micromanufacturing System

To evaluate the impact of each component optimization scheme on the overall performance of the machine, based on the optimized dimensions obtained for the spindle box, base, and column structural components, the components were assembled to generate the optimized overall machine model. Based on the finite element models of the machine before and after optimization, a comparative analysis of its static stiffness and dynamic characteristics was conducted. The results are presented below.

#### 3.2.1. Static Analysis

**Figure 9:**
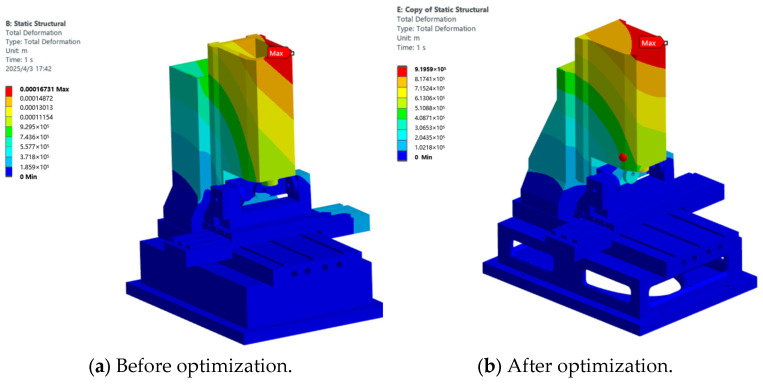
Cloud diagram of the total deformation of the whole machine.

**Figure 10:**
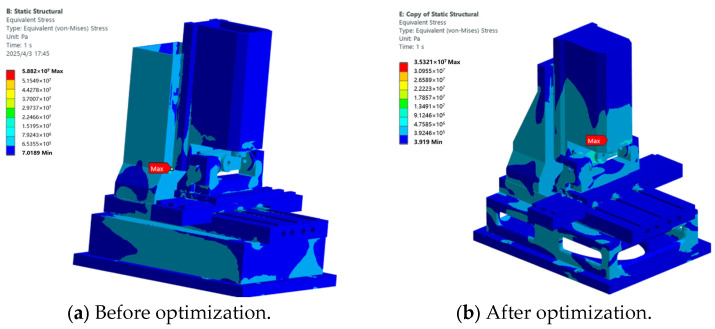
Stress cloud diagram of the whole machine.

Static analyses were conducted on the finite element models of the entire machine before and after optimization. The overall deformation maps of these models are shown in Figure 9a,b, with the maximum deformation decreasing from 0.17 mm to 0.09 mm. The stress maps of the entire machine before and after optimization are presented in Figure 10a,b, with the maximum stress dropping from 58 MPa to 35 MPa.

#### 3.2.2. Dynamic Analysis

The modal analysis was conducted on the finite element models of the entire machine before and after optimization, and the natural frequencies of each order of the entire machine before and after optimization were obtained. The first six natural frequencies are shown in Table 4. The first-order natural frequency increased from 50.68 Hz to 84.08 Hz, and the frequencies of the subsequent orders also increased simultaneously. The dynamic stiffness significantly enhanced. After optimization, the first six frequencies of the entire machine were all higher than those of the original structure, indicating an improvement in vibration suppression capability. The dynamic stiffness enhancement effect (84.08 Hz) meets the requirement of micro-processing systems for suppressing high-frequency vibrations [19], and is consistent with the research objective of precise control of piezoelectric micro-actuators.

## 4. Discussion

Although this study achieved relatively satisfactory optimization results, there are still certain limitations. Firstly, the optimization process mainly relies on finite element simulation and lacks physical experiment verification, which may affect the engineering accuracy of the results; secondly, some contact surfaces are simplified, and the model accuracy still needs to be improved; and thirdly, this study mainly focuses on static performance and modal response, and did not consider the performance under complex conditions such as thermal deformation, fatigue life, etc. Moreover, the geometries derived from topology optimization (Figure 7b and Figure 8b) serve as conceptual designs. Future work could employ size and shape optimization to further refine these geometries for even better performance and manufacturability.

To address these limitations, future research will be undertaken in the following directions: (1) Experimental validation using modal testing and deformation measurements on physical prototypes. (2) Implementation of thermal–structural coupling analysis to evaluate performance under thermal loads. (3) Development of fatigue life prediction methodologies integrated into the optimization framework. (4) Application of size and shape optimization techniques to enhance the manufacturability of topologically optimized designs.

## 5. Conclusions

Based on the existing finite element model of the entire machine, this paper proposes a multi-stage collaborative lightweight design process that combines sensitivity analysis, response surface method, and topology optimization. This process first screens the key components (the spindle box) using a sensitivity analysis, then uses the response surface method to perform multi-objective weight reduction optimization for the spindle box, maximizing the weight reduction while meeting the requirements of stress and deformation; finally, the topology optimization reconstruction is carried out for the machine base and the column, further reducing the mass without reducing the structural stiffness. The research conclusions are as follows:

(1) Significant weight reduction in key components: Under the premise of ensuring structural stiffness, the spindle box was reduced from 1379 kg to 590 kg (weight reduction of 57.2%), the base was reduced from 4624 kg to 2844 kg (weight reduction of 38.5%), and the column was reduced from 2210 kg to 1292 kg (weight reduction of 41.5%). The cumulative weight reduction in the entire machine exceeded 3000 kg, effectively reducing structural inertia and energy consumption.
(2) Static performance has significantly improved: The maximum static deformation of the entire machine has decreased from 0.17 mm to 0.09 mm, and the maximum stress has dropped from 58 MPa to 35 MPa. Meanwhile, the maximum stress values of all optimized components have also decreased. The stiffness has been significantly enhanced, meeting the requirements for structural stability in high-precision processing.
(3) Comprehensive optimization of dynamic characteristics: After optimization, the first six natural frequencies of the entire machine all increased. Specifically, the first mode frequency rose from 50.68 Hz to 84.08 Hz. The overall dynamic stiffness has been enhanced, and the vibration control and dynamic response capabilities were effectively improved.

In conclusion, the multi-objective collaborative optimization design method proposed in this study holds significant value both in theoretical research and in engineering practice. It not only provides an effective optimization framework for the lightweighting of ultra-precision micromanufacturing systems, but also offers reference value for structural optimization of precision micromanufacturing systems.

## Figures and Tables

**Table 1:** Comparison of maximum stress before and after optimization.

Weak Part	Before Optimization (MPa)	After Optimization (MPa)	Rate of Change (%)
Spindle box	61.79	18.09	−70.7
Base	4.68	4.31	−7.9
Column	10.16	4.17	−59.0

**Table 2:** Comparison of maximum deformation before and after optimization.

Weak Part	Before Optimization (mm)	After Optimization (mm)	Rate of Change (%)
Spindle box	0.166	0.091	−45.2
Base	0.019	0.008	−12.2
Column	0.123	0.067	−45.5

**Table 3:** Comparison of quality before and after optimization.

Weak Part	Before Optimization (kg)	After Optimization (kg)	Rate of Change (%)
Spindle box	1379	590	−57.2
Base	4624	2844	−38.5
Column	2210	1292	−41.5

**Table 4:** The first six orders of natural frequency of the whole machine.

Order	Natural Frequency Before Optimization (Hz)	Natural Frequency After Optimization (Hz)	Rate of Change (%)
First	50.68	84.08	65.9
Second	70.15	99.23	41.5
Third	123.71	158.05	27.8
Fourth	154.85	164.44	6.2
Fifth	172.51	193.69	12.3
Sixth	175.91	201.01	14.3

## Data Availability

The original contributions presented in this study are included in the article. Further inquiries can be directed to the corresponding author.
